# The Geriatric Nutritional Risk Index predicts sarcopenia in patients with cirrhosis

**DOI:** 10.1038/s41598-023-31065-1

**Published:** 2023-03-08

**Authors:** Chisato Saeki, Akiyoshi Kinoshita, Tomoya Kanai, Kaoru Ueda, Masanori Nakano, Tsunekazu Oikawa, Yuichi Torisu, Masayuki Saruta, Akihito Tsubota

**Affiliations:** 1 Division of Gastroenterology and Hepatology, Department of Internal Medicine, The Jikei University School of Medicine, Tokyo, Japan; 2 Division of Gastroenterology, Department of Internal Medicine, Fuji City General Hospital, Shizuoka, Japan; 3 Division of Gastroenterology and Hepatology, Department of Internal Medicine, The Jikei University Daisan Hospital, Tokyo, Japan; 4 Research Center for Medical Science, The Jikei University School of Medicine, Tokyo, Japan

**Keywords:** Gastroenterology, Medical research

## Abstract

Patients with cirrhosis are at high risk for sarcopenia and malnutrition, which are associated with reduced quality of life and increased mortality. We investigated the relationship between the Geriatric Nutritional Risk Index (GNRI) and sarcopenia/gait speed and assessed the usefulness of the GNRI for predicting sarcopenia in patients with cirrhosis. We evaluated 202 patients with cirrhosis and divided them into three groups based on baseline GNRI values: low (L)-GNRI (< 94.0, n = 49), intermediate (I)-GNRI (between 94.0 and 109.5, n = 103), and high (H)-GNRI groups (> 109.5, n = 50). Sarcopenia was diagnosed according to the criteria of the Japan Society of Hepatology. The prevalence of sarcopenia and slow gait speed was the lowest in the H-GNRI group (8.0% and 26.0%, respectively) and the highest in the L-GNRI group (49.0% and 44.9%, respectively). They increased stepwise with a decline in the GNRI group (*p* < 0.001 and *p* = 0.05, respectively). The GNRI values were significantly and positively correlated with handgrip strength, skeletal muscle mass index, and gait speed. Multivariate analysis identified lower GNRI as an independent risk factor for sarcopenia. The optimal cutoff value of the GNRI for predicting sarcopenia was 102.1 (sensitivity/specificity, 0.768/0.630). The GNRI was significantly associated with sarcopenia and physical performance and could be a helpful screening tool for predicting sarcopenia in patients with cirrhosis.

## Introduction

The liver plays a pivotal role in nutrient metabolism, and deterioration of the liver functional reserve leads to malnutrition. Patients with cirrhosis, especially decompensated cirrhosis, frequently have malnutrition, with the prevalence of 50–90%^1–4^. In such patients, reduced glycogen synthesis and storage and increased glycogenolysis promote gluconeogenesis from muscle-derived amino acids, leading to proteolysis and muscle loss^5^. Consequently, patients with cirrhosis complicated by malnutrition develop sarcopenia, defined as the progressive loss of skeletal muscle mass and function^6,7^. Sarcopenia is a critical risk factor for poor quality of life, mortality, and liver-related complications such as hepatic encephalopathy and infections^8–10^. Therefore, early diagnosis and appropriate therapeutic intervention for sarcopenia, such as nutrition and exercise therapy, are crucial for patients with cirrhosis. The European Working Group on Sarcopenia in Older People (EWGSOP) has adopted the SARC-F questionnaire as an initial screening tool for sarcopenia risk^11^. The SARC-F has high specificity for predicting sarcopenia without the use of specialized equipment; however, it has extremely low sensitivity in real-world clinical settings^12–15^. Therefore, it is desirable to establish a more sensitive screening method for sarcopenia.

The Geriatric Nutritional Risk Index (GNRI), which is calculated based on body weight and serum albumin level, was originally developed as a simple nutritional assessment tool to estimate the risk of morbidity and mortality in hospitalized older patients^16^. The GNRI scoring system classifies individuals into the following four nutrition-related risk groups, with lower GNRI scores indicating a higher risk of morbidity and mortality: major risk, GNRI < 82; moderate risk, 82 to < 92; low risk, 92 to ≤ 98; and no risk, > 98^16^. Intriguingly, in previous studies on patients undergoing hemodialysis, lower GNRI was a predictor of reduced muscle mass and strength and ability to walk^17–19^. Therefore, the GNRI may be a helpful screening tool for sarcopenia and physical performance, along with an assessment of malnutrition-related risk in patients with cirrhosis.

However, no studies have elucidated the relationship between the GNRI and sarcopenia/physical performance (gait speed) in patients with cirrhosis. This study aimed to examine this relationship and evaluate whether the GNRI is helpful for predicting sarcopenia in patients with cirrhosis.

## Results

### Patient characteristics

The baseline characteristics of the 202 patients enrolled in this study are presented in Table 1. The study cohort included 132 men (65.3%) and the median age was 69.0 (59.0–76.0) years. The frequencies of Child–Pugh class B/C (i.e., decompensated cirrhosis) and mALBI grade ≥ 2 were 31.7% (64/202) and 55.4% (112/202), respectively. The median GNRI value was significantly lower in patients with Child–Pugh class B/C than in those with Child–Pugh class A (i.e., compensated cirrhosis) (93.9 vs 106.2, *p* < 0.001; see Supplementary Fig. S1A online). Similarly, it was significantly lower in patients with mALBI grade ≥ 2 than in those with mALBI grade 1 (96.0 vs 108.2, *p* < 0.001; see Supplementary Fig. S1B online). The frequencies of sarcopenia and slow gait speed were 27.7% (56/202) and 36.1% (73/202), respectively.

**Table 1:** Comparison of clinical characteristics among the three groups based on the GNRI values.

Variable	ALL	L-GNRI	I-GNRI	H-GNRI	*p*-value
Patients, n (%)	202	49 (24.3)	103 (51.0)	50 (24.8)	
Men, n (%)	132 (65.3)	30 (61.2)	66 (64.1)	36 (72.0)	0.492
Age (years)	69.0 (59.0–76.0)	70.0 (59.0–76.5)	71.0 (63.0–76.0)	64.0 (53.0–73.0)	0.021
BMI (kg/m^2^)	23.6 (21.2–26.1)	20.6 (19.0–22.2)	23.2 (21.5–25.1)	27.8 (25.4–30.3)	< 0.001
Etiology					
HBV/HCV/Alcohol/other, n	18/67/64/53	0/18/17/14	13/33/33/24	5/16/14/15	0.290
Child–Pugh score	6 (5–7)	7 (6–8)	5 (5–7)	5 (5–5)	< 0.001
Child–Pugh A/B + C, n	138/64	16/33	74/29	48/2	< 0.001
ALBI score	− 2.53 (− 2.85– − 2.05)	− 1.85 (− 2.14– − 1.65)	− 2.56 (− 2.85– − 2.18)	− 2.85 (− 3.01– − 2.58)	< 0.001
mALBI grade (1/2a/2b/3)	90/40/68/4	5/3/37/4	48/25/30/0	37/12/1/0	< 0.001
GNRI	102.6 (94.0–109.5)	87.6 (80.8–91.1)	102.5 (97.6–106.2)	115.7 (111.0–120.7)	< 0.001
Total bilirubin (mg/dL)	0.9 (0.6–1.3)	1.0 (0.5–1.6)	0.9 (0.6–1.3)	0.9 (0.7–1.1)	0.966
Albumin (g/dL)	3.9 (3.4–4.2)	3.2 (2.8–3.5)	3.9 (3.6–4.2)	4.4 (4.0–4.5)	< 0.001
Prothrombin time (%)	82 (68–94)	78 (60–93)	82 (67–96)	85 (74–93)	0.163
eGFR (mL/min/1.73 m^2^)	64 (51–77)	65 (42–79)	65 (51–77)	61 (51–77)	0.999
M2BPGi (C.O.I)	3.06 (1.54–6.16)	5.64 (2.83–8.07)	2.99 (1.66–5.79)	1.66 (1.10–3.41)	< 0.001
BCAA (µmol/L)	398 (325–477)	340 (300–395)	402 (326–461)	463 (393–505)	< 0.001
Zinc (µg/dL)	63 (54–74)	54 (43–64)	64 (55–73)	73 (61–85)	< 0.001
Handgrip strength (kg)					
All patients	25.3 (18.5–34.9)	21.5 (16.6–28.8)	25.3 (19.1–34.0)	31.5 (22.5–41.0)	< 0.001
Men	31.6 (25.0–37.4)	25.6 (20.5–33.0)	31.6 (25.2–36.7)	37.8 (28.9–43.5)	< 0.001
Women	17.2 (14.4–22.3)	16.8 (14.6–20.0)	17.9 (14.5–22.5)	21.9 (12.9–23.2)	0.456
SMI (kg/m^2^)					
All patients	6.92 (5.95–7.85)	6.37 (5.36–6.94)	6.81 (5.96–7.63)	7.93 (6.95–8.73)	< 0.001
Men	7.34 (6.78–8.23)	6.82 (6.32–7.11)	7.33 (6.71–8.02)	8.22 (7.39–9.00)	< 0.001
Women	5.83 (5.20–6.39)	5.53 (5.05–6.11)	5.84 (5.08–6.34)	6.43 (5.80–7.46)	0.011
Sarcopenia, n (%)	56 (27.7)	24 (49.0)	28 (27.2)	4 (8.0)	< 0.001
Gait speed (m/s)	1.06 (0.85–1.25)	1.04 (0.75–1.21)	1.06 (0.86–1.21)	1.12 (0.99–1.35)	0.045
Slow gait speed, n (%)	73 (36.1)	22 (44.9)	38 (36.9)	13 (26.0)	0.144

Values are shown as median (interquartile range) or number (percentage). Statistical analysis was performed using the chi-squared test or the Kruskal–Wallis test, as appropriate. *ALBI* albumin-bilirubin, *BCAA* branched-chain amino acid, *BMI* body mass index, *eGFR* estimated glomerular filtration rate, *GNRI* Geriatric Nutritional Risk Index, *HBV* hepatitis B virus, *HCV* hepatitis C virus, *M2BPGi* Mac-2 binding protein glycosylation isomer, *mALBI* modified Albumin-Bilirubin, *SMI* skeletal muscle mass index.

### Clinical characteristics of the GNRI-based patient groups

**Figure 1:**
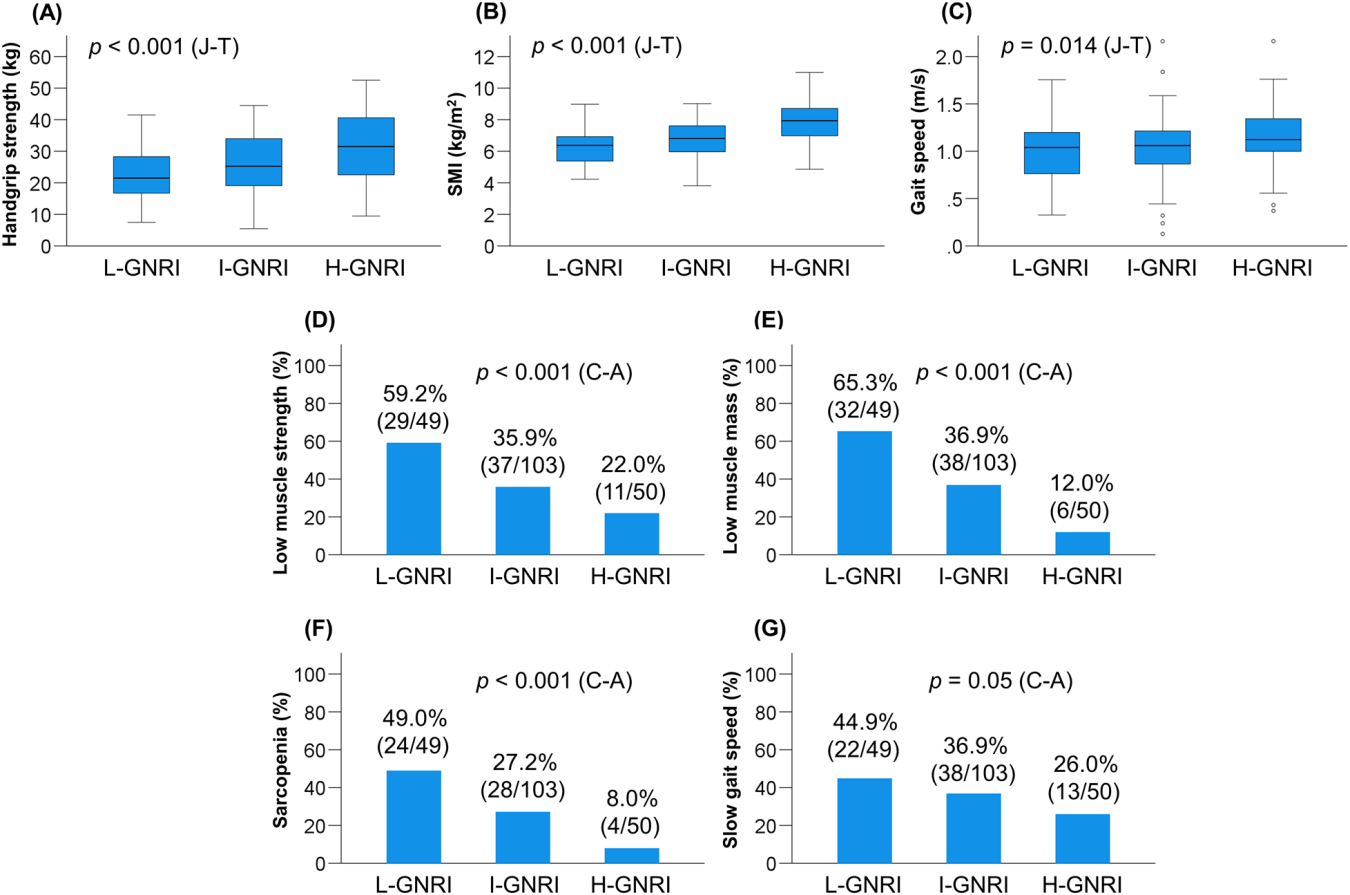
Comparison of clinical characteristics among the low-Geriatric Nutritional Risk Index (L-GNRI), intermediate-GNRI (I-GNRI), and high-GNRI (H-GNRI) groups. (**A**) Handgrip strength (*p* < 0.001), (**B**) skeletal muscle mass index (SMI; *p* < 0.001), and (**C**) gait speed (*p* = 0.014) significantly decreased stepwise with decreasing GNRI values. (**D**–**G**) The L-GNRI group had the highest prevalence of low muscle strength (59.2%), low muscle mass (65.3%), sarcopenia (49.0%), and slow gait speed (44.9%), while the H-GNRI group had the lowest prevalence of low muscle strength (22.0%), low muscle mass (12.0%), sarcopenia (8.0%), and slow gait speed (26.0%). A stepwise increase in the prevalence of these complication was observed with decreasing GNRI values (*p* = 0.05 for slow gait speed; *p* < 0.001 for all the rest). *J–T* Jonckheere–Terpstra test, *C–A* Cochran–Armitage test.

The proportions of L-GNRI, I-GNRI, and H-GNRI were 24.3% (49/202), 51.0% (103/202), and 24.8% (50/202), respectively (Table 1). There were significant differences among the three groups with regard to age (*p* = 0.021), Child–Pugh and ALBI scores (*p* < 0.001 for both), and M2BPGi, BCAA, and zinc levels (*p* < 0.001 for all). Of note, HGS (*p* < 0.001), SMI (*p* < 0.001), and gait speed (*p* = 0.014) decreased significantly in a stepwise fashion as the GNRI groups declined (Fig. 1A–C). Accordingly, the L-GNRI group had the highest prevalence of low muscle strength (59.2%), low muscle mass (65.3%), sarcopenia (49.0%), and slow gait speed (44.9%), whereas the H-GNRI group had the lowest prevalence of low muscle strength (22.0%), low muscle mass (12.0%), sarcopenia (8.0%), and slow gait speed (26.0%) (Fig. 1D–G). The prevalence of these sarcopenia-related complications increased in a stepwise fashion as the GNRI groups declined (*p* = 0.05 for slow gait speed; *p* < 0.001 for all the rest).

### Correlations between GNRI and sarcopenia-related factors

**Figure 2:**
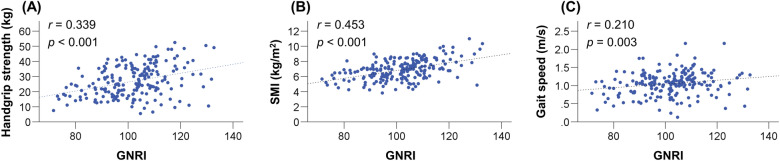
Correlations between Geriatric Nutritional Risk Index (GNRI) and handgrip strength, skeletal muscle mass index (SMI), and gait speed. GNRI values were significantly and positively correlated with (**A**) handgrip strength (*r* = 0.339, *p* < 0.001), (**B**) SMI (*r* = 0.453, *p* < 0.001), and (**C**) gait speed (*r* = 0.210, *p* = 0.003).

The GNRI values were significantly correlated with the following clinical factors: age, Child–Pugh score, ALBI score, PT, M2BPGi, BCAA, and zinc (Table 2). Notably, significant and positive correlations were found between the GNRI values and HGS (*r* = 0.339; 95% confidence interval [CI], 0.207–0.459; *p* < 0.001), SMI (*r* = 0.453; 95% CI, 0.332–0.559; *p* < 0.001), and gait speed (*r* = 0.210; 95% CI, 0.068–0.344; *p* = 0.003) (Fig. 2A–C).

**Table 2:** Correlations between the GNRI values and baseline characteristics.

Variable	Correlation coefficient (95% CI)	*p* value
Age (years)	− 0.148 (− 0.285 to − 0.007)	0.035
Child–Pugh score	− 0.590 (− 0.676 to − 0.489)	< 0.001
ALBI score	− 0.711 (− 0.775 to − 0.633)	< 0.001
Toral bilirubin	− 0.026 (− 0.167 to 0.117)	0.718
Prothrombin time	0.177 (0.036 to 0.311)	0.012
eGFR (mL/min/1.73 m^2^)	0.020 (− 0.122 to 0.162)	0.776
M2BPGi (C.O.I)	− 0.435 (− 0.545 to − 0.311)	< 0.001
BCAA (µmol/L)	0.407 (0.281 to 0.519)	< 0.001
Zinc (µg/dL)	0.497 (0.379 to 0.600)	< 0.001
Handgrip strength (kg)	0.339 (0.207 to 0.459)	< 0.001
SMI (kg/m^2^)	0.453 (0.332 to 0.559)	< 0.001
Gait speed (m/s)	0.210 (0.068 to 0.344)	0.003

*ALBI* albumin-bilirubin, *BCAA* branched-chain amino acid, *CI* confidence interval, *eGFR* estimated glomerular filtration rate, *M2BPGi* Mac-2 binding protein glycosylation isomer, *SMI* skeletal muscle mass index.

### Factors associated with sarcopenia

On univariate analysis, the following six variables were significant factors related to sarcopenia: age, etiology, Child–Pugh score, ALBI score, BCAA, and GNRI (see Supplementary Table S1 online). Finally, multivariate analysis revealed that advanced age (odds ratio [OR], 1.109; 95% CI, 1.062–1.157; *p* < 0.001), low BCAA (OR, 0.989; 95% CI, 0.984–0.994; *p* < 0.001), and low GNRI values (OR, 0.932; 95% CI, 0.895–0.970; *p* < 0.001) were significant and independent factors related to sarcopenia in patients with cirrhosis (Table 3).

**Table 3:** Significant factors associated with sarcopenia in patients with liver cirrhosis.

Variable	Univariate		Multivariate	
	OR (95% CI)	*p* value	OR (95% CI)	*p* value
Age (years)	1.077 (1.040–1.115)	< 0.001	1.109 (1.062–1.157)	< 0.001
Etiology	0.732 (0.526–1.020)	0.065		
Child–Pugh score	1.273 (1.016–1.593)	0.036		
ALBI score	1.999 (1.110–3.600)	0.021		
BCAA (µmol/L)	0.990 (0.986–0.994)	< 0.001	0.989 (0.984–0.994)	< 0.001
GNRI	0.920 (0.891–0.950)	< 0.001	0.932 (0.895–0.970)	< 0.001

*ALBI* albumin-bilirubin, *CI* confidence interval, *BCAA* branched-chain amino acid, *GNRI* Geriatric Nutritional Risk Index, *OR* odds ratio.

### Optimal cutoff values of age, GNRI, and BCAA for predicting sarcopenia

**Figure 3:**
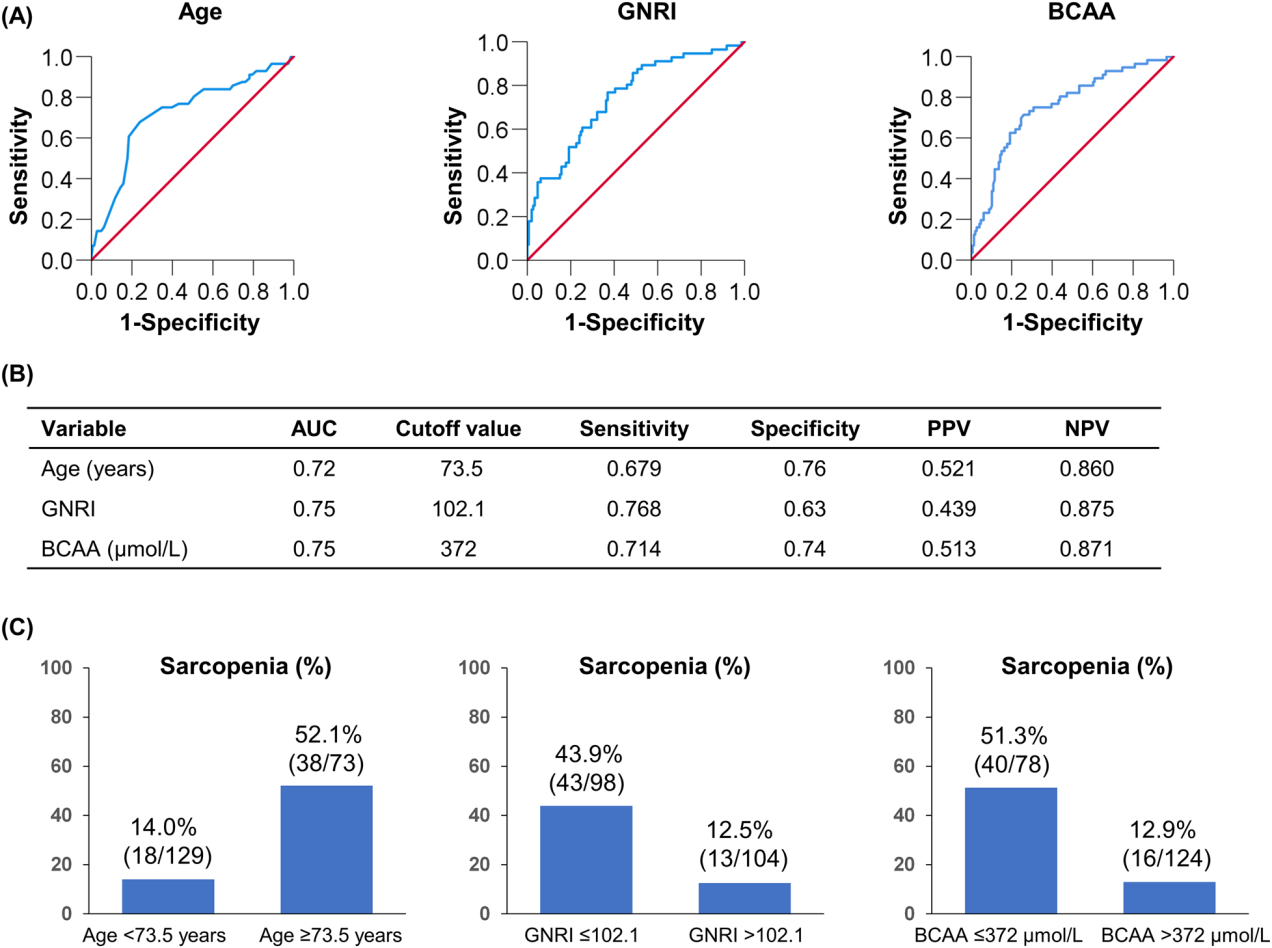
The receiver operating characteristic curve analysis for age, Geriatric Nutritional Risk Index (GNRI), and branched-chain amino acid (BCAA) in the prediction of sarcopenia. (**A**,**B**) The cutoff value for age was 73.5 years, with an area under the curve (AUC), sensitivity, and specificity of 0.72, 0.679, and 0.760, respectively. The cutoff value for GNRI was 102.1, with AUC, sensitivity, and specificity of 0.75, 0.768, and 0.630, respectively. (**C**) The cutoff value for BCAA was 372 µmol/L, with AUC, sensitivity, and specificity of 0.75, 0.714, and 0.740, respectively. (**C**) The prevalence of sarcopenia stratified by risk factors (age ≥ 73.5 years, Geriatric Nutritional Risk Index ≤ 102.1, and branched-chain amino acid ≤ 372 µmol/L). The prevalence of sarcopenia in each group was 52.1% (38/73), 43.9% (43/98), and 51.3% (40/78), respectively.

**Figure 4:**
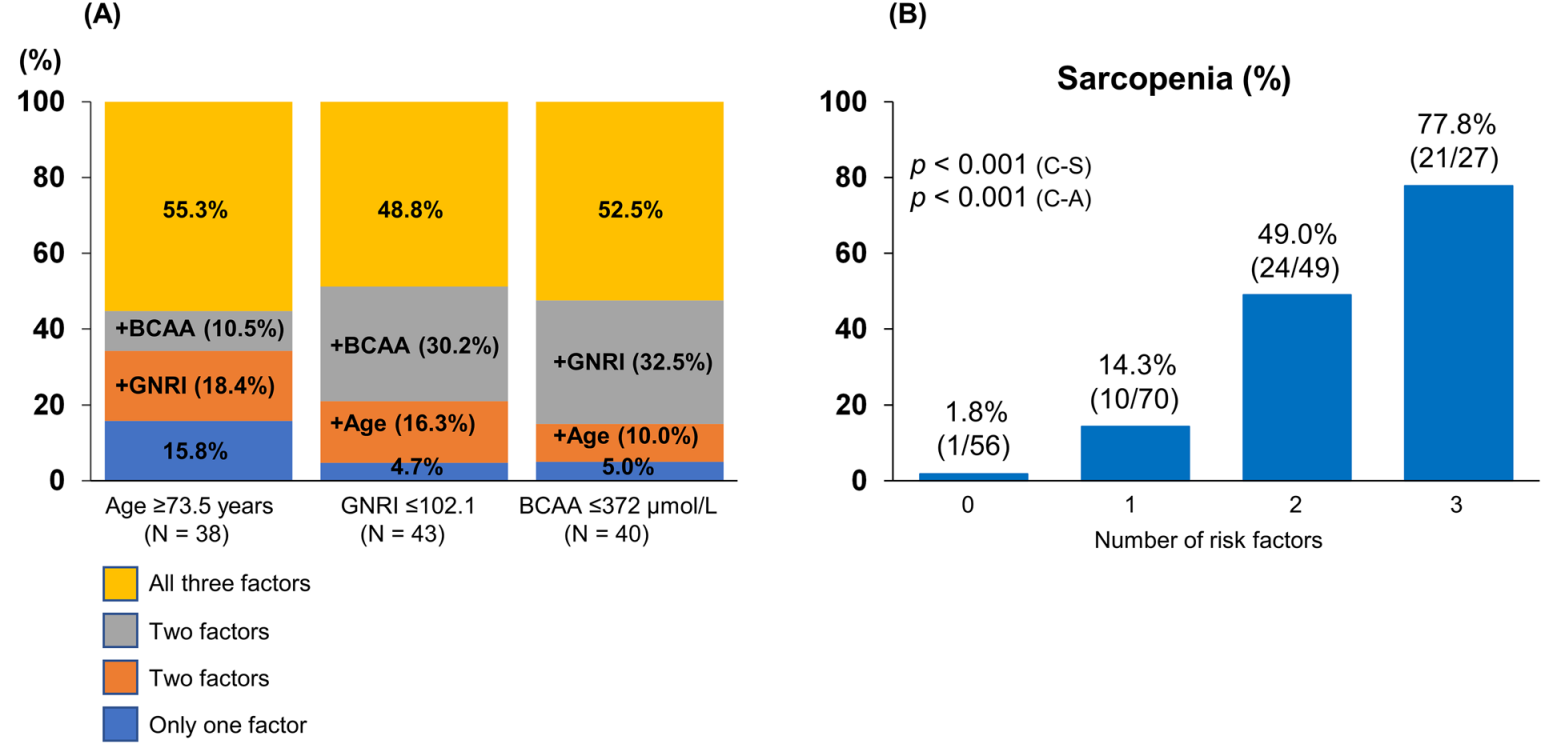
(**A**) Proportion of the number of risk factors in each risk group (age ≥ 73.5 years, Geriatric Nutritional Risk Index ≤ 102.1, and branched-chain amino acid ≤ 372 µmol/L). (**B**) The prevalence of sarcopenia in each group stratified by the number of risk factors. The prevalence of sarcopenia in the group with all three risk factors was the highest among the four groups (77.8% [21/27]; *p* < 0.001, adjusted residual = |6.2|), while the prevalence of sarcopenia in the group with no risk factors was the lowest (1.8% [1/56]; *p* < 0.001, adjusted residual = |5.1|), and its prevalence increased stepwise as the number of risk factors increased (*p* < 0.001). *C–A* Cochran–Armitage test; *C–S* Chi-squared test.

Figure 3 summarizes the cutoff values and diagnostic performances of age, GNRI, and BCAA for predicting sarcopenia. The cutoff values of age, GNRI, and BCAA were 73.5 years [area under the curve (AUC), 0.72; sensitivity/specificity, 0.679/0.760], 102.1 (0.75; 0.768/0.630), and 372 µmol/L (0.75; 0.714/0.740), respectively (Fig. 3A,B). The prevalence of sarcopenia was 52.1% (38/73), 43.9% (43/98), and 51.3% (40/78) in patients with age ≥ 73.5 years, GNRI ≤ 102.1, and BCAA ≤ 372 µmol/L, respectively (Fig. 3C). Some patients had two risk factors, while others had three risk factors (Fig. 4A). Therefore, we investigated changes in the prevalence of sarcopenia according to the number of risk factors (Fig. 4B). The group with all three risk factors had the highest prevalence of sarcopenia among the four groups (77.8% [21/27]; *p* < 0.001, adjusted residual = |6.2|), whereas the group with no risk factors had the lowest prevalence of sarcopenia (1.8% [1/56]; *p* < 0.001, adjusted residual = |5.1|; Fig. 4B). The prevalence of sarcopenia significantly increased stepwise as the number of risk factors increased (*p* < 0.001).

## Discussion

Malnutrition and sarcopenia are frequent complications that aggravate prognosis and quality of life and are serious health concerns in patients with cirrhosis^8–10^. Early assessment and therapeutic intervention for these complications are crucial. In the current study, we examined the relationship between the GNRI and sarcopenia-related components (muscle strength, muscle mass, and gait speed) and evaluated whether the GNRI is helpful for predicting sarcopenia in patients with cirrhosis. Notably, HGS, SMI, and gait speed significantly decreased stepwise with a decline in the GNRI-based groups and were significantly and positively correlated with the GNRI values. Accordingly, the prevalence of low muscle strength, low muscle mass, sarcopenia, and slow gait speed significantly increased stepwise with a decline in the GNRI-based groups. Multivariate analysis identified lower GNRI as a significant and independent factor related to sarcopenia. This is the first study to focus on the relationship between the GNRI and sarcopenia (muscle strength and muscle mass loss) and gait speed in patients with cirrhosis.

In one study of hospitalized older patients, the GNRI values were positively correlated with HGS and SMI, and could predict sarcopenia, with a GNRI cutoff value of 89.04^20^. In another study of patients undergoing hemodialysis, the high GNRI group (GNRI ≥ 100.8; mean, 103.1) had higher HGS and lean mass index than the low GNRI group (GNRI < 96.8; mean, 93.4)^17^. The GNRI was a significant and independent factor associated with independent walking ability, with a cutoff value of 86.7^17^. A recent study on patients with diabetes demonstrated that the low GNRI group (GNRI < 98; mean, 94.1) had a higher prevalence of sarcopenia than the high GNRI group (GNRI > 98; mean, 116.7)^21^. The former had higher levels of C-reactive protein than the latter, and the duration of diabetes (chronic inflammatory condition) was negatively correlated with the GNRI values. A previous study on patients with chronic kidney disease also revealed that GNRI was negatively correlated with the levels of interleukin-6 (proinflammatory cytokine)^22^. Increased levels of proinflammatory cytokines, such as interleukin-6 and tumor necrosis factor-α, promote proteolysis and cause sarcopenia through the activation of the ubiquitin–proteasome system^5^. These results suggest that low GNRI may be associated with chronic inflammatory status (e.g., cirrhosis, chronic kidney disease, and diabetes) and consequent loss of muscle mass and strength, and could be a good predictor of secondary sarcopenia.

The EWGSOP adopts the SARC-F questionnaire comprising the following five items as an initial screening tool for the assessment of sarcopenia: strength (S), assistance with walking (A), rising from a chair (R), climbing stairs (C), and falling (F)^11^. Every component has a score of 0–2, and a total score of ≥ 4 is suspected to have sarcopenia. Intriguingly, the SARC-F, like the GNRI, is associated with nutritional and inflammatory conditions in patients with gastrointestinal diseases^23,24^. One study reported that higher SARC-F scores were associated with moderate or severe malnutrition, as categorized using the controlling nutritional status score that is calculated from serum albumin level, total lymphocyte count, and total cholesterol level^23^. Another study reported that the SARC-F score had a positive correlation with the neutrophil to lymphocyte ratio, which reflects the inflammatory and immune status^24^.

However, a validation study of older Japanese adults showed that the SARC-F had high specificity (97.3%) but low sensitivity (8.0%) for identifying sarcopenia^13^. Similarly, a meta-analysis of seven studies, including 12,800 older adults, revealed high specificity (90%) but low sensitivity (21%) of the SARC-F^14^.

Meanwhile, another study of patients with chronic liver disease showed that modified SARC-F score of ≥ 1 had a higher discriminability for identifying sarcopenia than conventional SARC-F score of ≥ 4, with sensitivity and specificity of 65% and 68%, respectively^25^. Compared with the conventional SARC-F, the present study demonstrated that the GNRI could predict sarcopenia with lower specificity (63.0%) but considerably higher sensitivity (76.8%). Furthermore, the GNRI appears to yield higher sensitivity than the modified SARC-F score^25^. Given that the GNRI is calculated based on actual/ideal weight and serum albumin level, this index system is simple to apply and can be used even for individuals who have difficulty answering a questionnaire, such as those with dementia or an uncooperative attitude. Therefore, in clinical practice, the GNRI may be a convenient and suitable initial screening tool for sarcopenia.

The GNRI was originally established to estimate the risk of morbidity and mortality in hospitalized older patients (mean age, 83.8 years)^16^. The cutoff GNRI values for major, moderate, low, and no nutrition-related risks were < 82, 82 to < 92, 92 to ≤ 98, and > 98, respectively. Subsequent studies have demonstrated that the GNRI is useful for estimating the prognosis of patients with cancer, including hepatocellular carcinoma^26,27^. In elderly patients who underwent hepatectomy for hepatocellular carcinoma, the moderate- and major-risk groups (based on the original classification) were independent risk factors related to postoperative liver failure and severe complications^27^. It should be noted that the GNRI cutoff value of 102.1 for sarcopenia in this study cohort was higher than that of the original GNRI classification^16^ and the value of 89.04 for sarcopenia in hospitalized older patients (as described above)^20^. Given that the liver has multiple functions and plays a crucial role in nutrient metabolism, liver cirrhosis induces malnutrition, hypoalbuminemia, hyperammonemia, and body weight loss, and reduces IGF-1, vitamin D, testosterone, and BCAA levels, which are closely involved in the development of secondary sarcopenia^5,28,29^. Therefore, the GNRI cutoff value for sarcopenia in patients with cirrhosis appears to be higher than that for individuals without liver dysfunction. The GNRI-based assessment may be useful for introducing earlier nutrition and exercise therapy interventions to prevent the development of sarcopenia in patients with cirrhosis.

The present study had several limitations. First, we did not examine dietary intake, which may affect the GNRI and the development of sarcopenia. Second, this was a cross-sectional study; therefore, the morbidity and mortality were not evaluated. In the future, we will investigate the relationship between the GNRI and prognosis in patients with cirrhosis. Lastly, GNRI values may be overestimated in patients with ascites due to weight gain from ascites. However, this discrepancy was reduced in this study, since patients with massive ascites were excluded from this study.

## Conclusions

This study revealed that muscle strength, muscle mass, and gait speed are positively correlated with the GNRI and that the prevalence of sarcopenia and slow gait speed increases with a reduction in the GNRI, suggesting that the GNRI could be a convenient and helpful screening tool for sarcopenia in patients with cirrhosis. Appropriate nutrition-related risk assessment and early therapeutic intervention based on the GNRI may be useful for preventing sarcopenia or inhibiting disease progression in patients with cirrhosis.

## Methods

### Participants and study design

We enrolled 202 consecutive patients with cirrhosis who attended the Jikei University School of Medicine and Fuji City General Hospital between February 2017 and March 2021. This study cohort included 192 patients analyzed in our previous report^28^. Cirrhosis was diagnosed on the basis of laboratory tests and radiological imaging findings, including the presence of esophageal/gastric varices and ascites, and liver deformation and surface irregularities. Liver functional reserve was assessed according to the Child–Pugh classification and modified albumin-bilirubin (mALBI) grade^30,31^. The ALBI score was calculated using the following formula: ALBI score = (log 10 bilirubin [mg/L] × 17.1 × 0.66) + (albumin [g/dL] × 10 × − 0.085). The mALBI grading system classifies individuals into the following four groups: Grade 1, ≤ − 2.60; Grade 2a, > − 2.60 to − 2.27 ≤ ; Grade 2b, − 2.27 > to ≤ − 1.39; and Grade 3, > − 1.39, with Grade 3 being the most advanced liver disease^31^. Serum total bilirubin, albumin, estimated glomerular filtration rate (eGFR), prothrombin time (PT), zinc, branched-chain amino acid (BCAA), and Mac-2 binding protein glycosylation isomer (M2BPGi) were measured using standard laboratory methods. This study complied with the 2013 Declaration of Helsinki and was approved by the Ethics Committee of the Jikei University School of Medicine (approval no. 28-196) and Fuji City General Hospital (approval no. 156). Written informed consent was obtained from all the participants.

### Assessment of sarcopenia and gait speed

Sarcopenia was diagnosed according to the criteria advocated by the Japan Society of Hepatology (1st edition)^6^. The average handgrip strength (HGS) of the left and right hands was measured twice in a standing position using a digital Smedley-type hand dynamometer (T.K.K5401 GRIP-D; Takei Scientific Instruments, Niigata, Japan). In addition, the skeletal muscle mass index (SMI) was assessed using bioelectrical impedance analysis (BIA; InBody S10; InBody Japan, Tokyo, Japan). The cutoff values for decreased handgrip strength and SMI were < 26 kg and < 7.0 kg/m^2^ for men and < 18 kg and < 5.7 kg/m^2^ for women, respectively. Patients undergoing hemodialysis, with massive ascites, or implants were excluded due to the unreliability of the BIA method^7^. The 6-m walk was used to assess physical performance, with a slow gait speed defined as < 1.0 m/s.

### Patient grouping based on the GNRI values

The GNRI was calculated based on actual and ideal body weight and serum albumin values using the following formula: GNRI = (14.89 × albumin [g/dL]) + (41.7 × [actual body weight/ideal body weight])^16^. In the current study, the median GNRI value for all subjects was 102.6 (interquartile range, 94.0–109.5). Subjects were classified into three groups according to these first and third quartiles: low (L)-GNRI group, < 94.0 (first quartile); intermediate (I)-GNRI, between 94.0 and 109.5 (third quartile); and high (H)-GNRI group, > 109.5 (see Supplementary Fig. S2 online).

### Statistical analysis

Categorical variables are presented as numbers and percentages in parentheses. Continuous variables are presented as medians and interquartile ranges in parentheses. For categorical variables, the chi-squared test was used to evaluate the significance of group differences. For continuous variables, the Mann–Whitney *U* test and Kruskal–Wallis test were used to assess group differences, as appropriate. The Jonckheere–Terpstra test for continuous variables and Cochran–Armitage test for categorical variables were employed to evaluate whether significant trends were present among the groups. The Spearman’s rank correlation test was employed to evaluate the correlations between the GNRI and sarcopenia-related variables. Variables that reached *p* < 0.10 in univariate analysis were subsequently entered into multiple logistic regression analysis to identify significantly independent factors related to sarcopenia; however, BMI and albumin were excluded from multivariate analysis given that they are GNRI components. To estimate the optimal cutoff values for predicting sarcopenia, the area under the receiver operating characteristic (ROC) curve of age, GNRI, and BCAA was constructed. SPSS Statistics version 27 (IBM Japan, Tokyo, Japan) was used for each statistical analysis. Statistical significance was set at a *p*-value of less than 0.05.

## Supplementary Information

Supplementary Figure S1.

Supplementary Figure S2.

Supplementary Table S1.

Supplementary Legends.

## Data Availability

The data collected and analyzed in the current study are available from the corresponding authors on reasonable request.
