# Supplementary figures and images for: The Geriatric Nutritional Risk Index predicts sarcopenia in patients with cirrhosis
Source: Sci Rep. 2023 Mar 8;13:3888. doi: 10.1038/s41598-023-31065-1 (PMC9995649; doi:10.1038/s41598-023-31065-1)

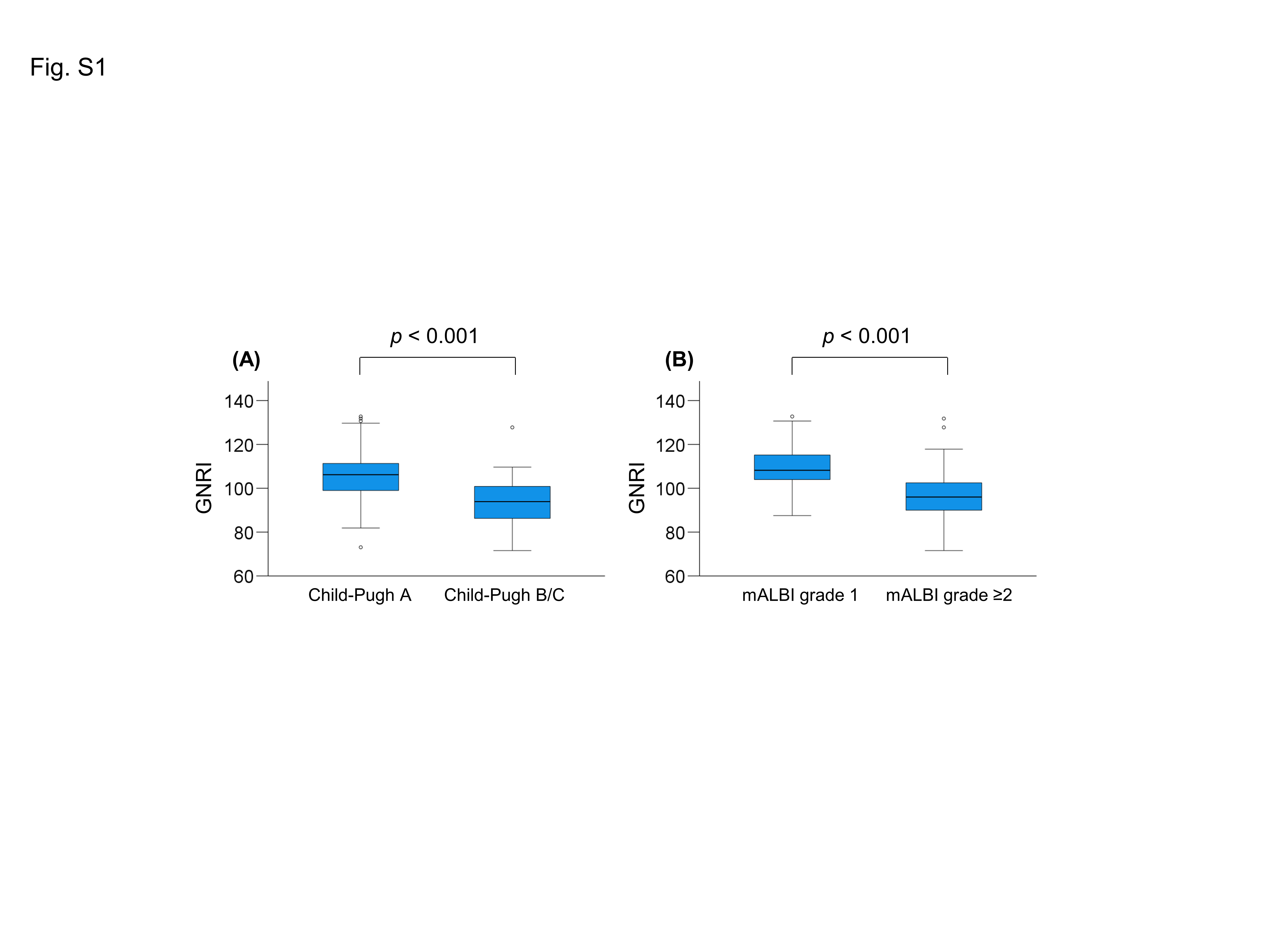

Supplement: Supplementary file 1 — Supplementary Figure S1. [file 41598_2023_31065_MOESM1_ESM.tif]

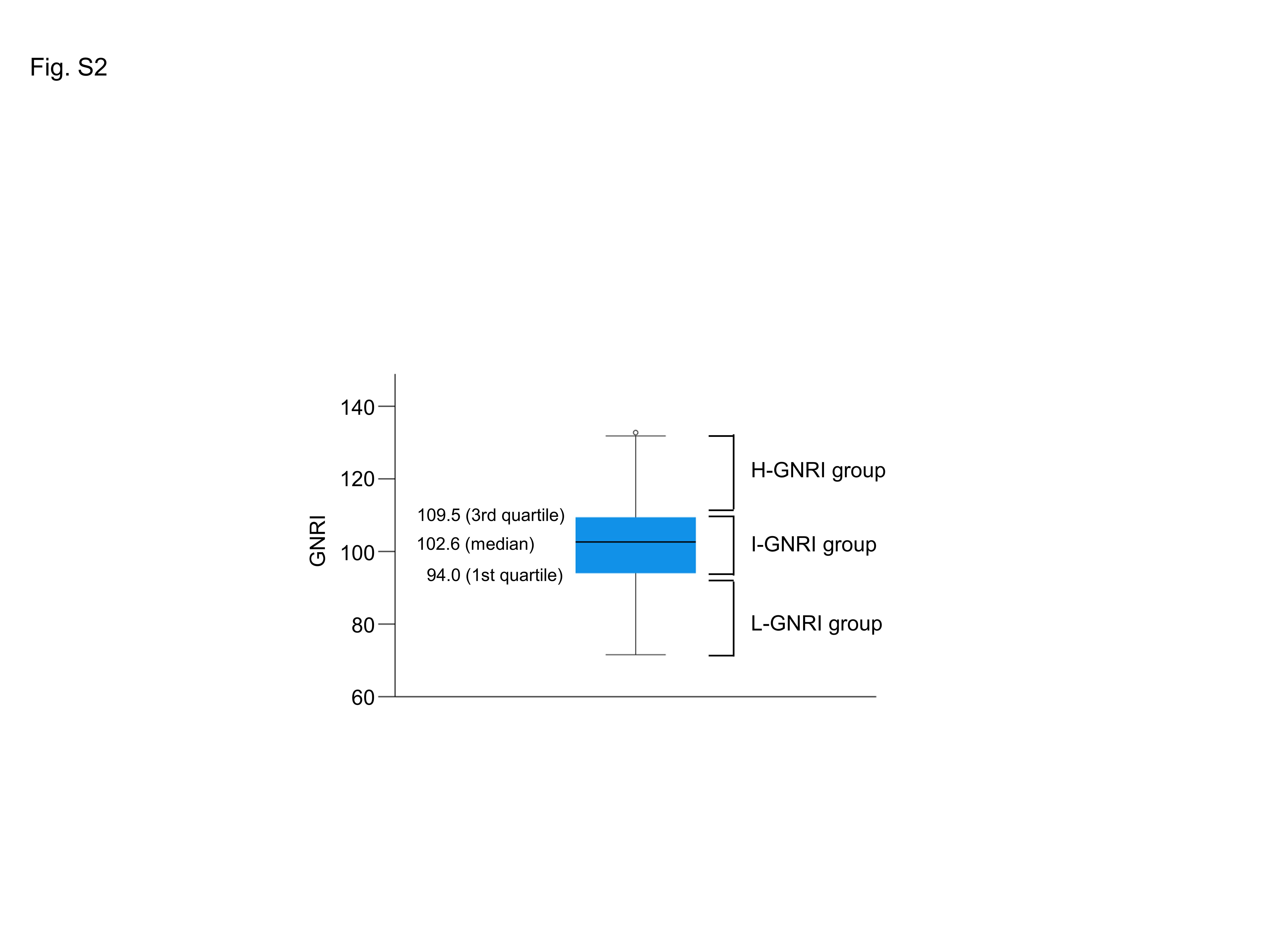

Supplement: Supplementary file 2 — Supplementary Figure S2. [file 41598_2023_31065_MOESM2_ESM.tif]
