# Supplementary material for: The Geriatric Nutritional Risk Index predicts sarcopenia in patients with cirrhosis
Source: Sci Rep. 2023 Mar 8;13:3888. doi: 10.1038/s41598-023-31065-1 (PMC9995649; doi:10.1038/s41598-023-31065-1)
Supplement: Supplementary file 3 — Supplementary Table S1. [file 41598_2023_31065_MOESM3_ESM.docx]

**Table S1. Univariate analysis of factors associated with sarcopenia**

| Variable | OR (95% CI) | *p* value |
| --- | --- | --- |
| Gender (Man) | 0.613(0.325–1.156) | 0.131 |
| Age (years) | 1.077(1.040–1.115) | < 0.001 |
| BMI (kg/m^2^) | 0.705(0.621–0.800) | < 0.001 |
| Etiology | 0.732(0.526–1.020) | 0.065 |
| Child-Pugh score | 1.273(1.016–1.593) | 0.036 |
| Child-Pugh B＋C | 1.288(0.672–2.469) | 0.446 |
| ALBI score | 1.999(1.110–3.600) | 0.021 |
| mALBI grade | 1.136(0.902–1.431) | 0.279 |
| GNRI | 0.920 (0.891–0.950) | < 0.001 |
| Total bilirubin (mg/dL) | 1.185(0.795–1.765) | 0.406 |
| Albumin (g/dL) | 0.451(0.257–0.794) | 0.006 |
| Prothrombin time (%) | 1.004(0.985–1.024) | 0.656 |
| eGFR (mL/min/1.73m^2^) | 0.988(0.973–1.003) | 0.127 |
| M2BPGi (C.O.I) | 0.999(0.922–1.083) | 0.986 |
| BCAA (µmol/L) | 0.990(0.986–0.994) | < 0.001 |
| Zinc (µg/dL) | 0.987(0.967–1.007) | 0.186 |

ALBI, albumin-bilirubin; BCAA, branched-chain amino acid; BMI, body mass index; eGFR, estimated glomerular filtration rate; GNRI, Geriatric Nutritional Risk Index; M2BPGi, Mac-2 binding protein glycosylation isomer; mALBI, modified Albumin-Bilirubin.
