# Supplementary material for: The Geriatric Nutritional Risk Index predicts sarcopenia in patients with cirrhosis
Source: Sci Rep. 2023 Mar 8;13:3888. doi: 10.1038/s41598-023-31065-1 (PMC9995649; doi:10.1038/s41598-023-31065-1)
Supplement: Supplementary file 4 — Supplementary Legends. [file 41598_2023_31065_MOESM4_ESM.docx]

**Figure S1.** Comparison of Geriatric Nutritional Risk Index (GNRI) values between groups stratified by Child-Pugh classification and modified albumin-bilirubin (mALBI) grade. (A) GNRI values were significantly lower in patients with Child-Pugh class B/C than in those with Child-Pugh class A (*p* < 0.001). (B) GNRI values were significantly lower in patients with mALBI grade≥2 than in those with mALBI grade 1 (*p* < 0.001).

**Figure S2.** Classification based on Geriatric Nutritional Risk Index (GNRI) values. The median (interquartile range) GNRI value was 102.6 (94.0–109.5). The 202 patients were classified into three groups: (1) the low-GNRI (L-GNRI) group with GNRI values <94.0 (first quartile); (2) intermediate-GNRI (I-GNRI) group with GNRI values between 94.0 and 109.5 (third quartile); and (3) high-GNRI (H-GNRI) group with GNRI values >109.5.
